# Heterogeneous Biomedical Database Integration Using a Hybrid Strategy: A p53 Cantcer Research Database

**Published:** 2007-02-20

**Authors:** Vadim Y. Bichutskiy, Richard Colman, Rainer K. Brachmann, Richard H. Lathrop

**Affiliations:** 1 Department of Computer Science.; 2 Department of Biomedical Engineering.; 3 Department of Medicine.; 4 Department of Biological Chemistry.; 5 Department of Pathology.; 6 Division of Hematology/Oncology.; 7 Institute for Genomics and Bioinformatics, University of California, Irvine, California 92697, U.S.A.

**Keywords:** Cancer, Data Warehousing, Heterogeneous Database Integration, Hybrid Database Integration, Mediation, p53

## Abstract

Complex problems in life science research give rise to multidisciplinary collaboration, and hence, to the need for heterogeneous database integration. The tumor suppressor p53 is mutated in close to 50% of human cancers, and a small drug-like molecule with the ability to restore native function to cancerous p53 mutants is a long-held medical goal of cancer treatment. The Cancer Research DataBase (CRDB) was designed in support of a project to find such small molecules. As a cancer informatics project, the CRDB involved small molecule data, computational docking results, functional assays, and protein structure data. As an example of the hybrid strategy for data integration, it combined the mediation and data warehousing approaches. This paper uses the CRDB to illustrate the hybrid strategy as a viable approach to heterogeneous data integration in biomedicine, and provides a design method for those considering similar systems. More efficient data sharing implies increased productivity, and, hopefully, improved chances of success in cancer research. (Code and database schemas are freely downloadable, http://www.igb.uci.edu/research/research.html.)

## Introduction

A long-held medical goal of cancer therapy is the discovery of small drug-like molecules able to rescue cancerous p53 mutants (Bullock and Fersht, 2001; Wang, Rastinejad, and El-Deiry, 2003; Bykov, Selivanova, and Kilman, 2003; Baroni et al. 2004; Brachmann, 2004). Toward this goal, different p53 data is produced by ten collaborating research laboratories within four Schools at the University of California, Irvine (UCI): *p53* cancer and suppressor mutations, and functional assays (in the School of Medicine) (Baroni et al. 2004; Dearth et al. 2006); bioinformatics and computational docking (Donald Bren School of Information and Computer Sciences) (Swamidass et al. 2005; Danziger et al. 2006); small molecule synthesis (School of Physical Sciences); and Nuclear Magnetic Resonance (NMR), X-ray crystallography, and other structural assays (School of Biological Sciences). This led to a need to develop a heterogeneous database that integrated each laboratory’s data.

Similar situations are very common in biomedical research and practice where multiple groups from diverse disciplines collaborate. This is especially true for complex diseases, such as cancer, where many interacting disciplines may be necessary for a successful attack. Each group often stores experimental data in a local, autonomous database. Consequently, related biomedical data is stored in multiple databases, which frequently have different schemas and are implemented with different technologies (Markowitz and Ritter, 1995). Database heterogeneities make access to information difficult (Sujansky, 2001).

Thus, multidisciplinary collaboration often gives rise to the need for heterogeneous databases. A heterogeneous database unites various databases, which have different schemas and technologies, by providing a uniform database schema and querying capabilities that integrate distributed data (Sujansky, 2001). The process of integrating data from multiple, autonomous, and heterogeneous sources is called heterogeneous database integration (Sujansky, 2001). Heterogeneous database integration is critical in biomedical discovery (Lacroix and Critchlow, 2003).

Heterogeneous database integration is a challenging topic, important in several application domains (Karasavvas et al. 2004), and is one of the most important computer science problems today (Zhou et al. 1995). It is especially difficult in biomedicine because biological data is inherently complex, in that (a) most rules have exceptions; (b) data is richly varied, from DNA and protein sequences to three-dimensional images to XML to flat files; and (c) there are complex associations between objects (Karasavvas et al. 2004).

This paper illustrates the hybrid database integration strategy as a viable approach to heterogeneous database integration in biomedicine, and suggests design principles for others considering similar projects. Here, the hybrid strategy was applied to a cancer informatics project involving p53 data from multiple laboratories. A global database schema was designed that captured each laboratory’s data. Heterogeneous p53 data sources were integrated by combining the mediator and the data warehouse approaches. More efficient data sharing implies increased productivity, and, hopefully, improved chances of success in cancer research.

### p53 Background

The central tumor suppressor protein p53 helps prevent uncontrolled cell growth. Upstream stress signals, such as DNA damage, lead to the activation of p53, which functions as a transcription factor for downstream genes involved in DNA repair, cell cycle arrest, and programmed cell death (apoptosis). Thus, p53 helps prevent a cell from propagating mutations due to DNA damage. If the *p53* gene is mutated or missing and the cell with damaged DNA continues to divide, cancer may result (Prives and Hall, 1992; Vogelstein, Lane, and Levine, 2000). In fact, it is estimated that close to 50% of human cancers have p53 inactivated due to gene mutations (Hollstein et al. 1991; Olivier et al. 2002). Furthermore, cancers with inactive mutant p53 are difficult to treat because they are especially resistant to radiation and chemotherapy (Wallace-Brodeur and Lowe, 1999; Soussi and Beroud, 2001).

## Database Background and Related Work

Heterogeneous database integration has been studied in the database research community for many years. However, no preferred solution or agreement of approach currently exists (Widom, 1996). In this section, first, the main challenges in heterogeneous biomedical database integration are described briefly. Then, we discuss approaches that have been proposed for heterogeneous database integration, including data warehousing, mediation, a less common hybrid strategy, and others.

### Challenges in Heterogeneous Biomedical Database Integration

Heterogeneous database systems are computational models and software implementations that provide a single, uniform query interface to data that are stored and managed in multiple, heterogeneous data sources (Sujansky, 2001). The goal of such systems is to provide database transparency to users as if the data were not distributed and all of the data sources were of the same type. Despite standardization efforts, it is believed in the research community that database heterogeneity will (and should) remain (because to prohibit heterogeneity would prohibit innovation). This is especially the case in the biomedical domain (Sujansky, 2001). Here, we summarize some of the main challenges involved in heterogeneous biomedical database integration.

*1) Query Models:* The core challenge of heterogeneous database integration, in biomedical and other domains, is that different data sources have different query models. A query model (Sujansky and Altman, 1994; Sujansky, 2001) is the model of data storage and information retrieval that a user of the database must know in order to retrieve data from it. A query model consists of the data model, database schema, query language, and data format. For example, biomedical data is variously stored in flat files, XML files, binary files, spreadsheets, and in relational, object-relational, and object-oriented databases (Chung and Wooley, 2003).

*2) Autonomous Data Sources:* Usually, biomedical data sources are autonomous (Chung and Wooley, 2003). This means that developers of the heterogeneous database do not have control over the data sources to be integrated. Each data source is free to modify its data and schema, and to restrict access to it (Karasavvas et al. 2004).

*3) Data Diversity:* Often, life science data to be integrated is very diverse, encompassing several research fields (Chung and Wooley, 2003; Hernandez and Kambhampati, 2004). This data ranges from flat files and literature publications, DNA and protein sequences, gene expression data, protein-protein interactions, molecular structures, and biomedical images to microarray chips, gels, light and electronic microscopy, NMR, and mass spectrometry. Furthermore, this data is often incomplete, inconsistent, and frequently updated. This diversity presents a major challenge in integrating biological data (Chung and Wooley, 2003).

*4) Representational Heterogeneity:* Similar data are often represented differently in different data sources. This representational heterogeneity consists of structural, naming, semantic, and content differences (Sujansky, 2001). Structural differences refer to schema differences. Naming (syntactic) differences occur when semantically equivalent objects (table and fields as well as data in fields) are named differently in different data sources. Semantic differences occur when names of objects in different data sources are similar or the same, but their meanings differ. Semantic ontologies may differ (McEntire et al. 2000). Content differences refer to differences in data between the data sources. The data may be implicit, derivable, or missing.

*5) Technical Heterogeneity:* These challenges occur due to differences in hardware platforms, operating systems, access protocols, transport formats, and programming languages between the data sources (Karasavvas et al. 2004).

### Data Warehousing

In data warehousing (Widom, 1995), data from each source is extracted, merged, and stored in a centralized repository (warehouse). The warehouse is a database with a global schema that combines the schemas of the sources. Queries on the system are evaluated at the warehouse without accessing the original sources. Client updates to the warehouse are usually not allowed since they are not propagated to the original sources and would make the warehouse inconsistent with the sources. Instead, the warehouse is updated from the data in the sources. There are multiple policies for updating the warehouse from the sources (Garcia-Molina, Ullman, and Widom, 2000).

Database integration based on data warehousing relies less on the network, and allows for improved query performance and optimization since queries are processed locally in the warehouse. Furthermore, since a local copy of the data is kept in a separate database from the data sources, data warehousing allows for data to be cleansed, annotated, and summarized (Widom, 1995; Hernandez and Kambhampati, 2004). The drawback of this approach is data duplication. This increases the cost of maintenance, the potential for data inconsistency, and the probability of outdated query results (Sujansky, 2001; Hernandez and Kambhampati, 2004). In general, data warehousing is suited for applications with predictable queries, applications requiring high query performance, and applications needing private copies of the data. Biomedical database integration systems that use the data warehousing approach include IGD (Ritter et al. 1994), GIMS (Cornell et al. 2001), GUS (Davidson et al. 2001), DoTS (http://www.allgenes.org/), Qu et al. (2002), and Tsou et al. (2006).

### Mediation

In mediation (Weiderhold, 1992; Domenig and Dittrich, 1999), a module called a “mediator” accepts a query from the client, determines the sources needed to answer the query, and decomposes the query into subqueries for each required source. The subqueries are translated to the source-specific query language via modules called “wrappers.” The results from the sources are translated back into the common query language by the wrappers. Finally, the mediator obtains results from the wrappers, combines them, and returns the final answer to the client. Mediation is the most common approach to data integration in biomedicine (Karasavvas et al. 2004).

Mediation can be query-centric (also known as global-as-view) or source-centric (also known as local-as-view) (Ullman, 1997; Li, 2001; Li, 2003; Hernandez and Kambhampati, 2004). In query-centric mediaton, users pose queries on the global views exported by the mediator. The mediator uses global views to expand user queries to queries on source data. In source-centric mediation, global predicates are used to construct source views and to pose user queries. The mediator uses source views to answer user queries. Ullman (1997) presents a detailed comparison of these two approaches to mediation.

The main advantage of mediation is that it reduces the maintenance required when data sources are modified or when new ones emerge. However, in contrast to data warehousing, mediation relies heavily upon the network, and can experience query performance degradation due to high network traffic, slow response, and unavailability of data sources (Sujansky, 2001; Hernandez and Kambhampati, 2004). Furthermore, the mediation approach is much more difficult to implement, and it is not feasible for data sources that do not support *ad hoc* queries. In general, mediation is appropriate for data that changes rapidly, for data with unpredictable queries, for sources that are reliable, and for queries on large amounts of data from many sources. Biomedical database integration systems that use the mediation approach include BioKleisli (Davidson et al. 1997), OPM Tools (Markovitz et al. 1999), TAM-BIS (Goble et al. 2001), K2 (Davidson et al. 2001), DiscoveryLink (Haas et al. 2001) and P/FDM (Kemp, Angelopoulos, and Gray, 2002).

### Hybrid Strategy

Voisard and Jürgens (1999) propose a hybrid strategy to data integration that combines data warehousing and mediation approaches. In the hybrid strategy, part of the data is queried on demand as in the mediation approach, but other data is extracted, filtered, and stored in the warehouse (Widom, 1996; Voisard and Jürgens, 1999). Ashish (2000) proposes a hybrid strategy as a way to improve the performance of a mediation approach by selectively importing data into the warehouse. In both (Voisard and Jürgens, 1999; Ashish, 2000), the warehouse is treated like a regular data source in a mediation approach. The hybrid strategy used in this paper is similar to the one implemented in Squirrel (Hull and Zhou, 1996). Squirrel implements a special mediator called a “Squirrel integration mediator” that queries part of the data on demand and stores other data in its materialized data storage. The disadvantage of Squirrel is the assumption that the underlying sources are databases.

Many large-scale database integration systems of the future will require both mediation and data warehousing (Widom, 1996; Voisard and Jürgens, 1999). While the best approach to data integration varies with the application, it is likely to be a hybrid strategy based on the combination of different approaches (Eckman, 2003). Such a hybrid strategy is less frequently discussed in the database literature compared to each approach alone, and this is even more true in biomedical database integration.

### Other Approaches

Federated database systems (Sheth and Larson, 1990; Garcia-Molina et al. 2000) integrate databases by implementing one-to-one connections between all databases that need to communicate with each other. Software components are written to translate queries between the databases. The disadvantage of this approach is that translation components need to be written for every pair of communicating databases. However, when limited communication between the databases is required, this approach may be the easiest to implement (Garcia-Molina et al. 2000).

Karasavvas et al. (2004) argue that agent technology is well suited for heterogeneous biomedical database integration as there are many similarities between multi-agent systems (MASs) and heterogeneous database systems based on the mediation approach. In this approach, Agent Communicating Languages (ACLs) act as common query languages, and ACLs and ontologies deal with technical and representational heterogeneities, respectively. Resource agents (RAs) act as wrappers in the mediation approach. In addition, the use of agent standards helps standardize database integration.

Philippi and Köhler (2004) propose an XML-based, ontology-driven approach based on data warehousing. First, the data sources to be integrated are converted from their native format into XML format. Then, these XML documents are inserted into a data warehouse, and are semantically defined against one or more ontologies (McEntire et al. 2000), which serve as semantic references. In addition, semantic definitions of data sources need to be generated. The authors’ motivation for using XML is that it is becoming a standard for data exchange in the life sciences (Achard, Vaysseix, and Barillot, 2001). To query the data warehouse, semantic query generation components are created that use the ontologies and semantic definitions of data sources. In this approach, syntactic and schematic differences between the data sources are resolved by using XML, and semantic differences are resolved through ontologies and semantic data source definitions. The advantages of this approach are that no integrated schema needs to be developed to provide integrated access to heterogeneous data sources, and changes in data sources do not affect the system. The disadvantages are that an XML database is needed for the data warehouse, an XML converter needs to be developed for each data source, and the quality of query results depends on the quality of the ontologies.

## Design Considerations

The design goal of the Cancer Research DataBase (CRDB) was to integrate data produced by collaborating research laboratories at the University of California, Irvine (UCI). The CRDB should allow researchers to share data, provide their results, and communicate efficiently, across laboratories in a common and convenient framework accessible through the Internet. However, the CRDB should not simply reproduce the sources’ data. The CRDB should store only the results that at least one laboratory feels would be interesting to other laboratories. In addition, the CRDB should not store all of the data features. It should store only the most important attributes of the data.

### Data Description

The CRDB integrated data that relates to experiments performed on small molecules with the goal of finding small molecules that restore function to p53 mutants and can be developed into new anti-cancer drugs. Computational docking is performed on a library of compounds to identify small molecules that have potential of restoring native function to p53 mutants. Each docking experiment is done on a specific docking target, here a p53 mutant. A docking target has multiple binding sites to which small molecules may bind. A molecule may have multiple conformations. The conformation of a molecule is its three-dimensional shape defined by angles of rotation about its bonds, as specified in a mol2 file. Docking uses computer algorithms to model how a molecule binds to a binding site of the target. The result of a docking experiment is a score that measures the ability of the molecule to bind the target’s binding site. Molecules with the best scores are synthesized. Finally, the synthesized molecules are assayed on the p53 mutants to determine what effect they have on the mutants.

### Design Method

Previously, a database containing *p53* cancer and suppressor mutations had been developed (“Mutants” data). We wanted to expand the database to contain the rest of the data (“Molecular” data). It was determined that it would be impractical simply to import the “Molecular” data into the database as in the data warehousing approach, and that the mediation approach was needed for a subset of the “Molecular” data. Therefore, we decided to implement a hybrid strategy to data integration by combining data warehousing and mediation approaches. Here, we describe the principles behind the design of the CRDB.

There are many ways to create a hybrid database architecture. It is a major design decision to determine what part of the data to import into the warehouse and what part to query on demand. In determining which approach to implement for a given data source, the designer needs to consider the characteristics of the data and the data source. Voisard and Jürgens (1999) discuss detailed general principles for the design of a hybrid strategy. Table 1 summarizes those principles used here.

In deciding which data integration approach to implement for each data source, it is possible to apply the principles in Table 1 in a qualitative manner by considering the requirements of the application and characteristics of the data source. However, as the number and complexity of data sources increase, qualitative design methods may become imprecise and inadequate. Instead, a more rigorous, quantitative method was applied in the design of the CRDB, which is shown in Table 2.

First, data sources were classified based on their characteristics as shown in Table 2A. Then, using the principles in Table 1, each characteristic was converted to the data integration approach that best implements that characteristic as shown in Table 2B. For example, data that changes periodically is best stored in a data warehouse while data that changes often is best accessed through mediation. Third, each approach was assigned a numerical value with warehouse = 1 and mediation = 0, and a design score was calculated for each data source by summing across the characteristics as shown in Table 2C. If a given data source had a score of 5 in Table 2C, it would be an ideal candidate for data warehousing; while if it had a score of 0, it would be ideal for mediation. In practice, most data sources are unlikely to be exactly ideal for either mediation or data warehousing; more likely, they would have some traits that favored one approach and other traits that favored the other approach. Thus, a median score of 2.5 was used to decide which approach to use for each data source. A data source with a score above the median was implemented with data warehousing, while one with a score below the median used mediation.

In theory, one wouldlike to use all of the principles in an optimal way. In practice, however, there are trade offs that need to be made between the principles. Mediation approach was chosen for computational docking and small molecule data even though query processing time may not be the best, because the size of the data and the frequency of data updates clearly favored this approach. On the other hand, implementing the data warehousing approach for computational docking and small molecule data would have improved query performance, but would have required frequent warehouse updates in order to maintain consistency of the warehouse and the high quality of query results.

## System Description

The CRDB was developed using Microsoft SQL Server 2000. The CRDB ran on IBM PC (Pentium) with a Microsoft Windows 2003 Server operating system. A global database schema was designed that captured all of the data. Computational docking and small molecules were integrated via connections to the research laboratories’ databases. Functional and structural assays were integrated by importing them into the CRDB. Connectivity between the CRDB and the web interface was provided by a ColdFusion application engine running on the server.

### Global Database Schema

**Figure 1.**
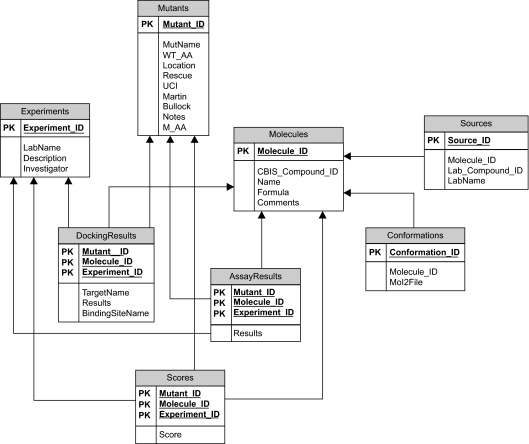
**CRDB global database schema.** The schema follows a design pattern with “Condition” tables (Molecules, Mutants, Experiments) related to “Results” tables (DockingResults and AssayResults). PK denotes a primary key.

A global database schema was designed that was able to store all of the data. The global database schema is shown in Fig. 1. Each molecule (“Molecules” table) may have more than one conformation (“Conformations” table) and it may come from more than one source (“Sources” table). There are two types of experiments (“Experiments” table) that are done on molecules: computational docking and biological assays. The results (“DockingResults” and “AssayResults” tables) of these experiments were captured in the database. Each type of experiment is done on a particular p53 mutant (“Mutants” table) and has a score (“Scores” table) associated with it.

The global schema design proved to be a difficult task. We had difficulty determining exactly what data needed to be integrated and how it ought to be stored. As a result, several design iterations were required. From the data, we identified two main results: docking and assay results. Each result was associated with different conditions: experiments, mutants and molecules. Thus, the schema followed a design pattern of “DockingResults” and “AssayResults” tables related to “Experiments,” “Mutants” and “Molecules” tables. This made the schema flexible to changes. If a new laboratory were to emerge that performed other experiments on molecules and mutants, a new “Results” table could be created with relationships to “Experiments,” “Mutants” and “Molecules” tables. Alternatively, if a new condition were to emerge, a new “Condition” table could be created with relationships to “DockingResults” and “AssayResults” tables.

### Hybrid Database Integration

**Figure 2.**
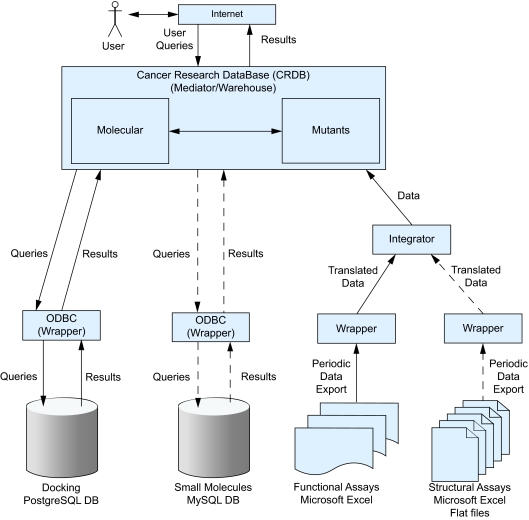
**System architecture and the hybrid strategy to data integration.** Docking and small molecule data use the mediation approach, while the functional and structural assay data use the data warehousing approach. The CRDB is both a mediator and a data warehouse. “Mutants” and “Molecular” are data marts of the warehouse. The ODBC drivers are wrappers in the mediation approach. Dashed lines indicate integration planned in the future.

The CRDB system architecture and the hybrid strategy to data integration are shown in Fig. 2. The CRDB included both a mediator and a data warehouse. The warehouse part of the CRDB consisted of two sub-warehouses (data marts) corresponding to the “Mutants” data and the “Molecular” data. Computational docking was integrated via a connection from the CRDB to the laboratory’s PostgreSQL database. In integrating computational docking data, we had two options: (1) import the data into the CRDB, or (2) connect from the CRDB to the laboratory’s database storing the computational docking data. Since the computational docking data was a large, frequently updated data set with millions of docking runs, we decided on the second option. The connection was implemented via the “Linked Servers” feature of Microsoft SQL Server. This was a convenient way of connecting two database servers, and was chosen for its availability and convenience. The ODBC driver for PostgreSQL acted as a wrapper that translated a query written in SQL Server into a query that PostgreSQL understands.

Functional assays were integrated by importing them intothe CRDB. We first imported functional assay data into a temporary table in the SQL Server database management system (DBMS) using the Data Transformation Services (DTS) feature of SQL Server. Because functional assay data was stored in Microsoft Excel spreadsheets, we needed to implement a wrapper that translated it from the spreadsheet format into the format and data model of CRDB. The wrapper was implemented as a data transformation stored procedure in SQL Server. Once translated, we again used DTS to insert functional assays into the CRDB. This approach to importing the functional assay data into the warehouse was chosen because of its availability and convenience within the SQL Server DBMS.

Computational docking and small molecule data were queried on demand by accessing the laboratories’ databases without importing them into the CRDB. Thus, for computational docking and small molecule data, the CRDB acted as a mediator. On the other hand, functional and structural assays were imported into the CRDB. In these cases, the CRDB acted as a data warehouse. Therefore, the overall strategy for heterogeneous database integration was hybrid.

The integration of small molecule and structural assay data is planned in the future. In integrating small molecule data, we will use the same approach as in computational docking. We will connect from the CRDB to the laboratory’s MySQL database via the “Linked Servers” feature. Similar to functional assays, structural assays will be integrated by first translating them from the native formats into the warehouse format, and then importing them into the CRDB via DTS.

## Results and Discussion

This paper presented the Cancer Research DataBase (CRDB) as an example of the hybrid strategy for heterogeneous biomedical database integration. The system architecture combined the data warehouse and the mediator within one entity. The CRDB was designed for p53 researchers in order to provide inter-laboratory communication for their efforts to discover small molecules that can restore native function to p53 cancer mutants. The researchers were able to query p53 mutants, functional assays, and computational docking data individually and in combination.

A researcher interactively specified a query through the selection of graphical user interface components. Depending on the query, the appropriate stored procedure was executed. For example, suppose a p53 researcher wishes to know with which p53 DNAbinding sites does the p53 mutant C141Y show activity, and which suppressor mutations (Baroni et al. 2004) rescue C141Y in the functional assay. The researcher selects the mutant name from the functional assay data drop down list, the stored procedure is executed that queries the functional data in the warehouse part of the CRDB, and the results are displayed.

For another example, suppose a p53 researcher wishes to know the results of docking studies done on p53 mutant G245S. In this case, after the researcher selects the mutant name from the docking data drop down list, the CRDB acts as a mediator by querying the docking laboratory’s PostgreSQL database. First, a query written as a SQL Server stored procedure is translated by the ODBC driver for PostgreSQL into a query that PostgreSQL understands. Then, the ODBC driver returns results from PostgreSQL to the CRDB. Finally, the results are displayed.

Developing a database that integrates data from different sources is a difficult task. Requirements analysis is essential. Multiple design iterations are inevitable. It is necessary to understand what data needs to be stored and its characteristics, what data is produced by each source, and what queries are to be performed on the database. It is also important to understand the characteristics of the data sources, such as how the data is stored, accessed, and updated locally. All of these factors need to be taken into account during the design of the global database schema and the overall system architecture.

In heterogeneous database integration, global schema design is the most critical, challenging, and time consuming phase. The design difficulty is compounded in that some sources do not have a database structure, and so store data in various software systems such as Microsoft Excel and flat files. This makes data integration more difficult because there is no standard method of connecting to the data.

When deciding which approach to implement for given data, the designer needs to consider the requirements of the application, the various parameters associated with the data and the data source (Voisard and Jürgens, 1999), and the various approaches to data integration including their strengths and weaknesses. For data that requires high query performance, or needs to be cleansed, annotated, or summarized, data warehousing is usually the best approach. Otherwise, mediation is an excellent choice since it may not require the development of a full global database schema.

Other important issues in heterogeneous database integration include data standards, ontologies, data annotation, and metadata. Data standards (also known as data exchange formats) may be used to help resolve schema and syntactic differences and to avoid developing multiple data transformation tools (Markowitz et al. 2003). In developing the CRDB, we did not impose a data standard because the sources had relatively small areas of overlapping content. However, in the future, as we integrate more data and more laboratories join the effort, a data standard may be suitable at least for the subset of the source data.

Ontologies are used to help resolve semantic differences and to facilitate querying across multiple data sources from diverse domains (McEntire et al. 2000; Chung and Wooley, 2003; Philippi and Köhler, 2004). In the current implementation, we did not find a great need for an ontology because the cooperating laboratories were largely consistent in their semantic usage. However, as the effort expands, an ontology may become necessary. Data annotation involves inferring additional information or knowledge about the data (Chung and Wooley, 2003; Hoebeke et al. 2005). In developing the CRDB, each laboratory provided all of the needed information about its data. Metadata, data describing data, facilitates more effective querying and interpretation of results. It is useful for the heterogeneous database to provide metadata on how the integrated data was generated and derived (Chung and Wooley, 2003).

Given the diversity of life science data, and the heterogeneous data sources in which this data is stored, there is no single “best” approach to heterogeneous database integration in biomedicine. The hybrid strategy is an attractive option. The goal of the hybrid strategy is to incorporate the strengths of multiple approaches while avoiding their weaknesses.

## Conclusions

This paper illustrated the hybrid strategy as a viable approach to heterogeneous database integration, one of the most important computer science problems today (Zhou et al. 1995), in a cancer informatics project involving p53. More examples are required to assess fully the advantages and disadvantages of hybrid database systems. Nonetheless, the hybrid strategy can provide a useful alternative system architecture for the designer facing the difficult choices that accompany heterogeneous database integration in biomedicine.

## Figures and Tables

**Table 1.** CRDB design principles.

**• Changes to data**
Mediation approach should be used for data that changes often. Data warehousing approach should be used for data that changes periodically.
**• Size of the data**
Mediation approach should be used for larger data. Data warehousing approach should be used for smaller data.
**• Availability of sources**
Mediation approach should be used for sources that are always available. Data warehousing approach should be used for sources that are often unavailable.
**• Required query processing time**
Mediation approach should be used for data with flexible timing constraints. Data warehousing approach should be used for data with stringent timing constraints.
**• Predictability of queries**
Predictable queries on data that does not change often should be written in advance with the results stored in the warehouse. In addition, knowledge of the queries to be performed on the system should be taken into consideration during global schema design.

**Table 2.** **CRDB design method. A.**Data sources were classified based on their characteristics. **B.** Each characteristic was converted to the data integration approach that best implements it. **C.** Each approach was assigned a numerical value with warehouse = 1 and mediation = 0, and a design score was calculated for each data source by summing across the characteristics.

A					
Data Source	Data Changes	Data Size	Source Availability	Timing Constraints	Query Predictability
Docking	often	large	sometimes	flexible	low
Small Molecules	often	large	sometimes	flexible	high
Functional Assays	periodically	small	sometimes	flexible	high
Structural Assays	periodically	small	sometimes	flexible	low

B					
Data Source	Data Changes	Data Size	Source Availability	Timing Constraints	Query Predictability
Docking	mediation	mediation	warehouse	mediation	mediation
Small Molecules	mediation	mediation	warehouse	mediation	warehouse
Functional Assays	warehouse	warehouse	warehouse	mediation	warehouse
Structural Assays	warehouse	warehouse	warehouse	mediation	mediation

C						
Data Source	Data Changes	Data Size	Source Availability	Timing Constraints	Query Predictability	Design Score
Docking	0	0	1	0	0	1
Small Molecules	0	0	1	0	1	2
Functional Assays	1	1	1	0	1	4
Structural Assays	1	1	1	0	0	3
